# Effectiveness of perampanel for focal seizures determined by interictal gamma oscillation regularity analysis

**DOI:** 10.1002/epi4.13033

**Published:** 2024-08-21

**Authors:** Junya Okabe, Yosuke Sato

**Affiliations:** ^1^ Department of Neurosurgery Showa University School of Medicine Tokyo Japan; ^2^ Brain Function Analysis and Digital Medicine Research Institute Showa University Tokyo Japan

**Keywords:** epilepsy, focal epilepsy, focal seizure, gamma oscillation regularity, perampanel

## Abstract

Although perampanel (PER) has received approval as an antiseizure medication, reports quantifying its antiseizure effects using electroencephalography (EEG) remain scarce. In a previous study, we demonstrated that the interictal high gamma oscillation regularity (GOR) on scalp EEG is an excellent marker of epileptogenicity. Herein, we investigated whether the antiseizure effect of PER could be quantified through interictal GOR analysis of scalp EEG data. To investigate this, we examined the interictal GOR from 20 s of scalp EEG data before and after PER administration collected from five patients with epilepsy with focal seizures. Prior to PER administration, each patient presented with localized areas with high GOR consistent with brain lesions or seizure semiology. In all patients, the seizures improved following PER administration, and the localized high GOR, which is considered an epileptogenic focus, disappeared. These results indicate that interictal GOR analysis may be a useful tool for the quantitative assessments of the antiseizure effects of PER in focal epilepsy.

**Plain Language Summary:**

This study explored whether perampanel (PER)'s antiseizure effects can be quantified using interictal high gamma oscillation regularity (GOR) analysis from scalp EEG data. Analyzing 20‐second EEG segments before and after PER administration in five patients with focal epilepsy, we found that high GOR areas, indicative of epileptogenic foci, disappeared following PER administration. The results suggest that interictal GOR analysis could effectively quantify the antiseizure effects of PER.

**Key points**

- Interictal high gamma oscillation regularity (GOR) on scalp EEG is a marker of epileptogenicity in focal epilepsy.
- We found that the localized high GOR, considered an epileptogenic focus, disappeared after perampanel (PER) administration.
- Interictal GOR analysis can be used to assess antiseizure effects of PER in focal epilepsy.

## 1 INTRODUCTION

It is now widely accepted that epileptic seizures can be caused by glutamate‐mediated neuronal hyperexcitability.^1^, ^2^ Perampanel (PER), an antagonist of the α‐amino‐3‐hydroxy‐5‐methyl‐4‐isoxazolepropionic acid (AMPA) receptor, is a new antiseizure medication. PER is currently licensed in Japan as both a monotherapy and an adjunctive therapy in the treatment of partial‐onset seizures (with or without secondarily generalized seizures) in patients with epilepsy aged 4 years and older; and as an adjunctive therapy for primary generalized tonic–clonic seizures among patients with epilepsy aged 12 years and older. Several studies have previously applied quantitative electroencephalography (EEG), which is commonly used to evaluate the pharmacological effects of central nervous system drugs, to determine changes caused by PER and to investigate the relationship between these EEG changes and adverse effects.^3^, ^4^, ^5^ However, the results of these reports do not necessarily reveal any changes in EEG in cases of decreased seizures, and there are currently no reports that consider EEG as an indicator of antiseizure effects.

We previously demonstrated the utility of gamma oscillation regularity (GOR) analysis of interictal EEG data in the evaluation of the epileptogenic zone in focal epilepsy.^6^, ^7^ Indeed, there is evidence to show that high interictal GOR may reflect the epileptic synchronous activities of interneurons associated with focal epileptogenicity,^8^, ^9^, ^10^ and is an excellent marker of epileptogenic zone localization.^6^, ^7^ This study aimed to investigate the possibility of comparing interictal GOR before and after PER administration to quantitatively evaluate the antiseizure effectiveness of PER.

## 2 METHODS

### 2.1 Patients

This study involved the analysis of five patients with focal epilepsy treated in our outpatient department. All patients met the following criteria: (1) focal epilepsy clinically diagnosed based on seizure semiology; (2) PER monotherapy started at 2 mg orally before bedtime, then increased by 2 mg at intervals of at least 2 weeks, with a maintenance dose of 4 mg once daily; and (3) no concomitant use of antiseizure medications other than PER. All procedures were performed by a board‐certified epileptologist. This study was approved by the Research Ethics Board of the Showa University School of Medicine. All procedures were performed in accordance with the latest version of the Declaration of Helsinki.

### 2.2 EEG recordings

EEG data were recorded using a Nihon Kohden EEG system at 16 electrode sites (Fp1, Fp2, F3, F4, C3, C4, P3, P4, O1, O2, F7, F8, T3, T4, T5, and T6), in accordance with the international 10–20 system, with the two ear lobes jointly forming the reference. EEG signals were recorded at a sampling rate of 500 Hz, a 1–60 Hz bandpass filter, and a time constant of 0.3 s. A 60‐Hz notch filter was applied to all channels. The 20‐second EEG data for the GOR analysis were inspected to ensure that they were not contaminated by artifacts. For each patient, EEG data were obtained at the time point prior to PER administration, and 3–6 months following the start of the 4 mg PER maintenance dose.

### 2.3 Interictal GOR analysis

The selected interictal 20‐second EEG data were downsampled to 200 Hz, where the timescale factor, *τ*, in multiscale entropy analysis was set at 3–7, corresponding to the gamma frequency (28.6–66.7 Hz). We defined the GOR as the average score with *τ* = 3–7 and calculated the GOR for each 20‐second EEG epoch. We then calculated the mean and standard deviation (SD) of the measured GOR for each patient. *Z* values were calculated using the following equation: *Z* = (individual GOR − mean GOR)/(SD of GOR). Calculations were performed for each electrode in each patient. Lower *Z* values indicated a higher GOR. To visually assess the GOR, we created a color‐coded GOR (opposite *Z* values) superimposed on the scalp EEG electrodes. The detailed algorithm for the interictal GOR analysis using the sample entropy method has been described in our previous studies.^6^, ^7^

## 3 RESULTS

The characteristics of each patient are summarized in Table 1. A total of five patients (mean age [±SD], 34.8 ± 16.1 years; 4 males and 1 female) were examined. The seizures were considered to be of temporal lobe origin in three patients, and frontal lobe origin in three patients. In terms of outcomes after PER administration, three patients showed >50% response, while two achieved seizure resolution.

**TABLE 1:** Patient characteristics.

Patient #	Sex	Age	MRI findings	Seizure type	Outcome after PER
1	M	Young adulthood	Right temporal postoperative scar	Focal to bilateral tonic–clonic seizure	>50% response
2	F	Middle age	Left amygdala enlargement	Focal impaired awareness seizure	Seizure‐free
3	M	Young adulthood	Left hippocampal sclerosis	Focal impaired awareness seizure	>50% response
4	M	Middle age	Left frontal hemorrhagic scar	Focal motor seizure	>50% response
5	M	Young adulthood	Right frontal cavernoma	Focal motor seizure	Seizure‐free

Abbreviations: MRI, magnetic resonance imaging; PER, perampanel.

**FIGURE 1:**
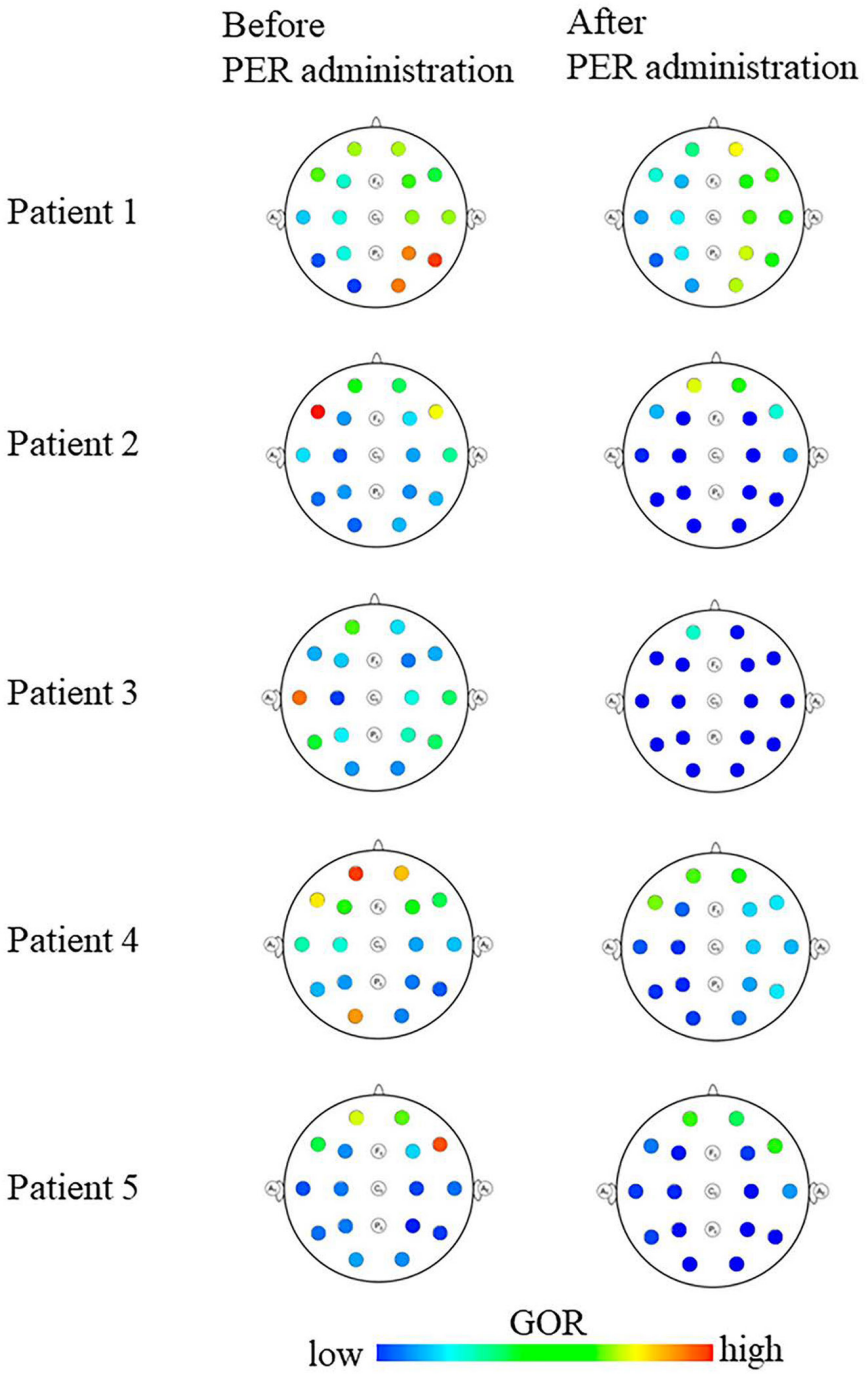
Results of interictal gamma oscillation regularity (GOR) analysis before and after perampanel (PER) administration. In all patients, the high GOR regions before PER administration disappeared following PER administration.

Prior to PER administration, all patients showed localized areas with high GOR, which were epileptogenic zones consistent with a brain lesion and/or the patient's seizure semiology. In all patients, the seizures improved following PER administration, and the localized high GOR disappeared (Figure 1).

## 4 DISCUSSION

This study showed that the epileptogenic zone visualized by interictal GOR analysis disappeared following PER administration in focal epilepsy. This is consistent with the fact that PER administration clinically improves seizures. Lanzone et al. previously found that patients who qualified as drug responders showed increased alpha power both at baseline and following PER titration, indicating that the alpha power could be a prognostic marker of PER responsiveness^3^; however, it does not allow direct quantitative assessment of PER antiseizure efficacy. Similarly, in studies using quantitative EEG, Ahn et al. and Liguori et al. showed that PER administration had little negative effect on cognitive function^4^, ^5^; however, these studies did not demonstrate the antiseizure effect of PER using EEG data. As such, we believe that this study is the first to demonstrate the antiseizure effect of PER using interictal GOR analysis of scalp EEG data.

One prior study using high‐frequency oscillations of somatosensory evoked potentials (SEP‐HFOs) by Lanzone et al. reported that the introduction of PER as an add‐on therapy reduced the area of total HFOs, acting primarily on the early burst related to the thalamocortical pathways. Furthermore, the P24/N24 amplitude in SEP, which seems to reflect a form of cortico‐subcortical integration, was shown to increase before PER administration, normalizing after PER administration.^11^ These results suggest that PER may exert a diffuse action in relation to its AMPA‐blocking effect on cortical–subcortical hyperexcitability. Our interictal GOR analysis also showed a trend toward an overall decrease in GOR following PER administration compared to before, indicating an AMPA‐blocking effect on cortical–subcortical hyperexcitability in the brain as a whole.

AMPA receptor activation in the postsynaptic membrane by glutamate, an excitatory neurotransmitter, is strongly involved in focal ictogenesis. PER inhibits this activation, thereby suppressing neuronal hyperexcitability and synchronization, and subsequently exerting antiseizure effects.^1^, ^2^ As interictal GOR analysis may detect such neural synchrony in the epileptogenic zone as localized high GOR,^6^, ^7^, ^8^, ^9^, ^10^ we speculated that the pharmacological antiseizure effects of PER could be observed directly from scalp EEG data. This further suggests that PER may make it difficult to achieve epileptogenicity (i.e., prevent intractable epilepsy).

The primary limitation of this report is that it investigated only a small number of cases in which only PER was administered, and therefore did not include cases in which other antiseizure medications were concomitantly administered; further research to address these limitations is currently ongoing.

## 5 CONCLUSION

Interictal GOR analysis may be a useful tool to quantitatively evaluate the antiseizure effects of PER in focal epilepsy.

## AUTHOR CONTRIBUTIONS

Sato was involved in conception and design, analysis and interpretation of data, study supervision, administrative/technical/material support, statistical analysis and approved the final version of the manuscript on behalf of all authors. Okabe and Sato drafted and critically revised the article. All authors reviewed the submitted version of manuscript and involved in acquisition of data.

## FUNDING INFORMATION

None.

## CONFLICT OF INTEREST STATEMENT

The authors declare that they have no known competing financial interests or personal relationships that could have appeared to influence the work reported in this paper. We confirm that we have read the Journal's position on issues involved in ethical publication and affirm that this report is consistent with those guidelines.

## Data Availability

The raw data supporting this article will be made available by the authors without undue reservation.
